# Long-acting family planning method switching among revisit clients of public health facilities in Dire Dawa, Ethiopia

**DOI:** 10.1186/s40834-016-0028-z

**Published:** 2016-09-14

**Authors:** Meselu Atnafe, Nega Assefa, Tadesse Alemayehu

**Affiliations:** 1 Dire Dawa City Administration Health Bureau, Dire Dawa, Ethiopia; 2 College of Health and Medical Sciences, Haramaya University, Dire Dawa, Ethiopia

**Keywords:** Method switching, Long-acting contraceptives, Long-acting family planning method, Contraception

## Abstract

**Background:**

“Contraceptive switching” from one method to another is a common phenomenon. Switching from a more effective long-acting method to a less effective method exposes women for unplanned pregnancy. The aim of this study was to assess the level and factors associated with long-acting family planning method switching to other methods.

**Method:**

A facility-based cross-sectional study was conducted from January to March 2013 on 634 women attending public health facilities in Dire Dawa City Administration, Ethiopia. Participants of the study were revisit clients of family planning service and were interviewed as they appear in the clinics. Data were analyzed using crude and adjusted logistic regression, and results were reported using OR and corresponding 95 % CI.

**Results:**

Long-acting family planning method switching among revisit clients was 40.4 %; switching from implant was 29.8 % and from IUCD, it was 10.6 %. The main reasons for methods switching were side effects of the methods such as bleeding, weight loss, and feeling of arm numbness. The tendency of switching was less among married women (AOR = 2.41, 95 % CI: 1.01, 5.74), women who had 2–4 and 5 and more children (AOR 3.00, 95 % CI: 1.59, 5.67) and (AOR 2.07, 95 % CI: 1.17, 3.66), respectively. It was also less among women who want to stop birth (AOR 5.11, 95 % CI: 1.15, 24.8), among those who mentioned health care providers as source of information for family planning (AOR 1.88, 95 % CI: 1.18, 3.01), and among women whose husbands were aware of their use of the methods (AOR 3.05, 95 % CI: 1.88, 4.94).

**Conclusions:**

Method switching from long-acting contraceptives to less effective methods is high. Method switching was significant among unmarried women, who had one child, plan to postpone fertility, and whose husbands were not aware of their wive’s use of the method. In the provision of family planning service, the health care providers should give adequate information about each method and risks of method switching. Appropriate family planning Information Education and Communication (IEC) and Behavioral Change Communication (BCC) strategies should be emphasized.

## Background

The term “contraceptive switching” refers to the case where a person changes method of birth control [1]. Many family planning users switch from long-acting contraceptive to other methods. Long-acting contraceptives, such as implant and intrauterine contraceptive device are convenient for users and have high effectiveness in preventing pregnancy [2]. Despite the advantages, long-acting contraceptive methods remain not the primary choice among clients and the uptake of the method is minimal [3–6]. In many developing countries, majority of current contraceptive users had used at least one other method; such as oral contraceptives, injectable and other methods in the past. An exception is Cambodia, where only a quarter of current users have switched from other methods. Countries with the highest rates of contraceptive switching are Gabon and Colombia, where more than 80 % of current users have used another method before [7].

Switching from a more effective method to a less effective method (long-acting to short-acting contraceptives) creates a period for unplanned pregnancy. During the early period of use, as clients are still learning how to use the new method accurately and consistently, the risk of unplanned pregnancy is high [1]. Unplanned pregnancy increases the risk of unsafe abortion and STIs/HIV infection [8, 9]. Currently, more than 200 million women in developing countries want to avoid pregnancy but they cannot do it for many reasons. Some of the major obstacles are lack of access to proper information and proper health care services, contraceptive failure, contraceptive discontinuation, opposition from husbands, misperceptions about side effect and cost [10–14].

Though family planning enables couples to control their fertility, during method switching, the risk of adverse effects and failure to adapt the new method results in unplanned pregnancy [15]. So far method switching has received little attention from researchers and health executive bodies, hence, the size of the problem is not clearly known.

The aim of the study was to assess the level and factors contributing to method switching from long-acting contraceptives to other methods in Dire Dawa city, Ethiopia. Identification of the levels of method switching and factors related to switching will help researchers and health executive bodies at national and regional level to guide programs, lead proper implementation and evaluate the outcomes of interventions.

## Methods

The study was conducted in Dire Dawa City Administration, Eastern Ethiopia from January to March 2013. Dire Dawa is a city located about 525 km from Addis Ababa, the capital of Ethiopia. The total population of the city is 342,827 of which 233,224 (67.93 %) lives in urban and 108,610 (32.07 %) in the rural areas [16]. The city has five hospitals (1 public, 3 private and 1 company), 16 health centers (9 in urban and 7 in rural), 31 health post, 9 kebeles, 38 peasant associations, and two non-governmental clinics (Marie Stopes and FGAE). Family planning is given in all public and private health facilities integrated with the routine health system in maternal and child health (MCH) clinics [17].

A facility based cross-sectional study using quantitative and qualitative techniques was conducted. The sample size for the quantitative study was 634. In this study, data was generated from four health centers and one public hospital. Facility client registration was used as a sampling frame and systematic random sampling was used to identify specific women for interview. Only revisit women were invited for interview. To support and supplement the quantitative findings, four Focus Group Discussions (FGDs) consisting of 8–12 participants were conducted. Participants of the FGDs were selected on the basis of prior use of long-acting methods and their willingness to share their experiences.

The quantitative data was collected using pre-tested structured questionnaire adapted from Family Health International (FHI) 2009 questions. Open-ended discussion guide was used to lead the discussion of the focus groups. Female high school graduates collected the data and two nurses supervised the data collection process. A one-day training on the objective of the study, data collection tools and interview methods was given for the research team by the principal investigator.

Quality of data was maintained through adequate training of study tool, continuous follow-up during data collection, on-site supervision, and pre-test on one of the nearby health centers. In addition, data was carefully checked for completeness and consistency on daily basis. Each questionnaire was assigned a unique code to maintain its anonymity and facilitate data entry. Any confusion on the data collection procedure and/or responses was handled in a timely fashion.

Frequencies and odds ratios with 95 % confidence intervals (CI) were used to describe the results and show the presence of association between independent and dependent variable. Multiple logistic regression was used to control the effect of confounders. Qualitative data were tape recorded, transcribed verbatim and hand summarized using summary sheet. Notes taken during interviews also helped to complete the template.

The study was approved by the Institutional Research Ethical Review Committee (IRERC) of College of Health and Medical Sciences, Haramaya University. Official letter of cooperation was obtained from Dire Dawa Town Health Bureau. Participants were briefed about the purpose, procedures, potential risk, benefits, and right of participants to withdraw from the study at any time. Before the start of data collection, written informed consent was obtained from study participants. Confidentiality and privacy of information were maintained throughout the research process.

## Results

A total of 634 women participated in the interview with a response rate of 100 %. These women were the ones who came for the second time to the family planning service. Among the 634 participants, the majority, 536 (84.5 %), were urban residents. The age of respondents ranged between 15 and 45 with mean age of 26.2 years (SD = ±5.7 years), the median age was 25 years. Regarding the educational status, 42.6 % had primary schooling and 32.6 % can read and write. By their occupation, the majority, 57.7 %, of respondents were housewives and 62.1 % were Muslims (Table 1).

**Table 1:** Socio-demographic characteristics of re-visit clients at family planning clinics in public health facilities, Dire Dawa City Administration, 2013 (*n* = 634)

Socio-demographic characteristics	Frequency	Percent
Age of the women		
15–19	62	9.8
20–24	189	29.8
25–29	181	28.5
30–34	127	20.0
35+	75	11.8
Educational status of the women		
Canot read and write	207	32.6
Primary (1–8 grade)	270	42.6
Secondary (9–12 grade)	117	18.5
Diploma and above	40	6.3
Educational status of husband		
Canot read and write	120	18.9
Primary (1–8 grade)	198	31.2
Secondary (9–12 grade)	213	33.6
Diploma and above	103	16.3
Religion of the women		
Muslim	394	62.1
Orthodox	208	32.8
Protestat	29	4.6
Catholic	3	0.5
Occupation of the women		
House wife	366	57.7
Government employee	77	12.1
House maid	11	1.7
Merchant	59	9.3
Student	42	6.6
Farmer	35	5.5
Daily laborer	39	6.1
Others	5	0.8
Marital Status of the women		
Single	36	5.7
Married	577	91.0
Divorced	14	2.2
Widowed	7	1.1

The majority, 577 (91 %), of the respondents were married and 555(87.5%) had ever given birth. Among those who had ever given birth, 196 (30.9 %) had at least one child, 289(45.6 %) had 2 to 4 children, and 70(11 %) have 5 and more children. At the time of the interview, nearly 50 % of the respondents would like to have an additional child (Table 2).

**Table 2:** Fertility characteristics of re-visit clients to family planning clinics in public health facilities, Dire Dawa City Administration, 2013

Fertility characteristics	Frequency	Percent
Ever given birth (*n* = 634)		
No	79	12,5
Yes	555	87,5
Number of children (*n* = 555)		
1	196	30,9
2–4	289	45,6
5+	70	11,0
Intention to have more children (*n* = 555)		
Yes	320	50,5
No	184	29,0
Depends on husband	22	3,5
Depends on God	18	2,8
I am not sure	11	1,7

**Fig. 1:**
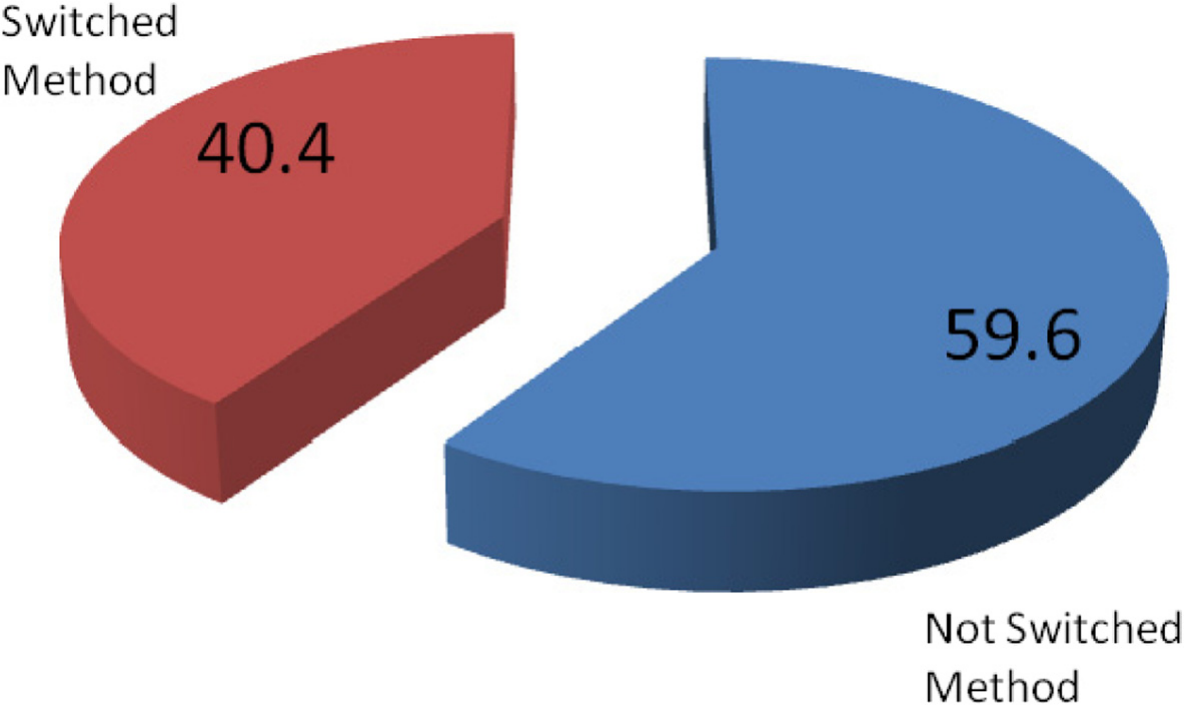
Long-acting family planning method switching among revisit clients at public health facilities in Dire Dawa city Administration, 2013 (*n* = 634)

**Fig. 2:**
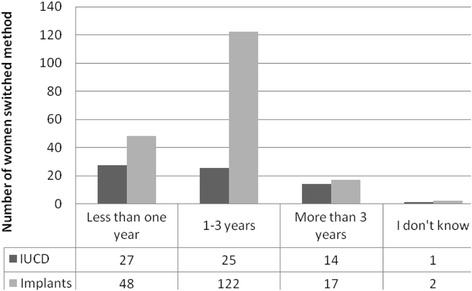
Duration of use of long-acting family planning before switching to other method among revisit clients at public health facilities in Dire Dawa city Administration, 2013(*n* = 634)

Of the total participants, 498(78.5 %) were using injection and 136(21.5 %) were using oral contraceptive pills. Level of switching from long-acting family planning methods to other methods was 40.4 %. Switching from implants was reported by 189(29.8 %) respondents, while the rest 67(10.6 %) switched from Intrauterine Contraceptive Device (IUCD). Regarding the timing of method switching, 75(11.8 %) switched during the past 12 months and 143 (23.2 %) switched during 24–36 months (Figs. 1 and 2).

**Fig. 3:**
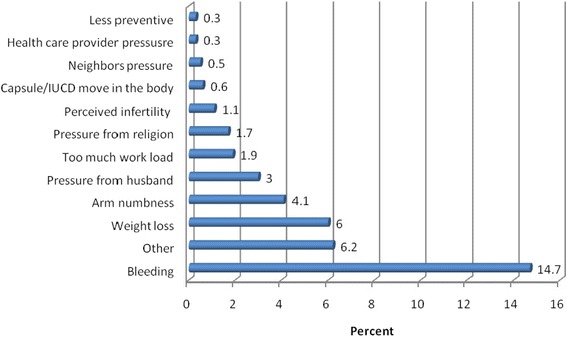
Reasons for switching long-acting family planning methods at public health facilities in Dire Dawa city Administration, 2013 (*n* = 634)

Regarding the reasons for method swiching; bleeding, weight loss and feeling of numbness were the major problems while using the previous long-acting family planning method. FGD participants responded that side effects of the methods were major reasons for shifting method (Fig. 3).

Regarding, sources of information of family planning, the majority, 305 (48.1 %) responded health care providers were the main source, followed by neighbors 89(14 %), and Health Extension Workers (HEWs) 83(13.1 %). Regarding the person who chose family planning method for the women, 474 (74.8 %) participants responded, they themselves chose the method (Table 3).

**Table 3:** Family planning related characteristics of re-visit client at public health facilities in Dire Dawa City Administration, 2013(*n* = 634)

Family planning related characteristics	Frequency	Percent
Where do you get information about family planning?		
Husband	32	5.0
Neighbors	89	14.0
Health care providers	305	48.1
Health Extension Workers	83	13.1
Others	125	19.7
Is your husband aware about your use of family planning?		
No	95	15.0
Yes	539	85.0
Who chooses family planning method for you?		
Myself	474	74.8
Husband	96	15.1
Neighbors	2	0.3
Health care provider	61	9.6
Other	1	0.2
Do religious leader in your area consider family planning?		
Yes	120	18.9
No	354	55.8
I donot know	160	25.2
Which family planning methods religious leaders considered? (*n* = 120)		
Pills	5	8
Injectable	22	3.5
Implant	1	0.2
IUCD	1	0.2
Calander method	11	1.7
Abstinence	6	0.9
All methods	52	8.2
Short methods only	22	3.5

Marital status showed significant association with method switching; married women were three times less likely to switch method (COR = 2.96, 95 % CI: 1.27, 6.85). Women who had 2–4 children were less likely to change long-acting method to other method (COR = 1.63, 95 % CI: 1.12, 2.48), and women who had more than 5 children were 3.8 times less likely to switch long-acting method than those who had one child (COR = 3.8, 95 % CI: 2.14, 6.74). Women who want no more pregnancies were 5.2 times less likely to switch long-acting method compared to those women who need spacing (COR = 5.2, 95 % CI: 1.10, 24.9) (Table 4).

**Table 4:** Predictors of long acting family planning method switching among re-visit clients in public health facilities, Dire Dawa City Administration, 2013(*n* = 634)

Characteristics	Switched method			
	No (%)	Yes (%)	Crude OR (95 % CI)	Adjusted OR(95 % CI)
Age group				
15–19	50(80. 7)	12(19.3)	1.00	1.00
20–24	134(70.9)	55(29.1)	1.71(0.85,3.46)	0.38(0.73,2.01)
25–29	99(54.7)	82(45.3)	3.5(1.72, 6.91)*	0.68(0.13,3.56)
30–34	63(49.6)	64(50.4)	4.2 (2.06, 8.69)*	0.74(0.14,3.93)
35^+^	32(42.7)	43(57.3)	5.7(2.60,12.67)^*^	1.05(0.19,5.75)
Marital status				
Unmarried	29(80.6)	7(19.4)	1.00	1.00
Married	349(58.4)	249(41.6)	2.96(1.27,6.85)	2.41(1.01,5.74)^*^
Number of children				
1	133(67.8)	63(32.2)	1.00	1.00
2–4	163(56.4)	126(42.6)	1.63(1.12,2.48)^*^	3.00 (1.59, 5.67)^*^
5+	25(35.7)	45(64.3)	3.8(2.14, 6.74)^*^	2.07(1.17, 3.66)^*^
Future fertility control				
To space birth	207(64.7)	113(35.3)	1.00	1.00
To stop birth	85(46.2)	99(53.8)	5.2(1.10,24.92)^*^	5.11(1.15,24.79)^*^
Depends on husband	11(50.0)	11(50.0)	4.5(0.79,25.77)	3.48(0.59,20.48)
Depends on God	10(55.6)	8(44.4)	3.6(0.60,21.61)	3.95(0.64,24.34)
I am not sure	9(81.8)	2(18.2)	2.5(0.52, 11.66)	3.08(0.64,14,84)
Source of family planning information				
Husband	19(59.4)	13(40.6)	1.00	1.00
Neighbors	56(62.9)	33(37.1)	1.76(0.79, 3.94)	1.82(0.80,4.15)
Health Extension Workers	161(52.8)	144(47.2)	1.52(0.85, 2.71)	1.31(0.71,2.39)
Health care providers	52(62.6)	31(37.4)	2.30(1.56,3.61)^*^	1.88(1.18, 3.01)^*^
Others	90(72.0)	35(28.0)	1.53(0.85,2.86)	1.03(0.55,1.94
Husband is aware about use of Family planning				
Yes	345(64.0)	194(36.0)	1.00	1.00
No	33(34.7)	62(65.3)	3.34(2.11,5.28)^*^	3.05(1.88,4.94) ^*^

N.B. where P-value < 0.05 and ^*^ Adjusted for all variables in the table

Results of multiple logistic regression showed that, married women were 2.4 times less likely to switch method compared to unmarried women (AOR = 2.41, 95 % CI: 1.01, 5.74). Women who had 2–4 children were 3.0 times less likely to switch long-acting method than those who have one child (AOR = 3.00, 95 % CI: 1.59, 5.67). Similarly, women who had more than 5 children were 2.07 times less likely to switch long-acting method than those who have one child (AOR = 2.07, 95 % CI: 1.17, 3.66). Women who want no more pregnancies were 5.1 times less likely to switch method compared to those who wish to have more children (AOR = 5.11, 95 % CI: 1.15, 24.8). Women who got initial information about family planning from health care providers were 2 times less likely to switch compared to those women who received information from their husband (AOR = 1.88, 95 % CI = 1,18,3.01). On other hand, women whose husbands were aware about their use of long-acting family planning method were 3.05 times less likely to switch compared to those whose husbands were aware of their use of the method (AOR = 3.05, 95 % CI = 1.88–4.94) (Table 4).

## Discussion

Long-acting Family Planning method switching among revisit clients was 40.4 %. The major determining factors for method switching were age of the respondent, educational status of husband/partner, marital status, number of children, and desire for having more children. The main reasons for method switching from long-acting method to other methods were bleeding, weight loss, and numbness in the arm.

Participants in this study were selected only from public health facilities, as majority of family planning users in the city are visiting public health facilities. Yet, there are some women who receive service from private and NGO clinics, the views of these women are not reflected in this report. Similarly, the perspectives of husband/partner and health care provider were not included. Hence readers are advised to read the paper taking these into account.

Method switching was 40.4 %, and switching from implant was 29.8 % and the rest, 10.6 %, switched from IUCD. This is relatively low compared to reports from other studies. For example, in Egypt method switching from IUCD and Norplant to another method was 45.9 % [18]. Similar study from 14 developing countries showed that there were high rates of switching in Peru (70.5 %) and Morocco (69.8 %). In Vietnam, two-thirds of women who had an IUCD reported to switch to other methods (n = 306/434) [19]. In contrast to our findings, method switching is low in Bolivia (16.7 %) and Kazakhstan (25.2 %). Reports of USAID in 2007, indicated that high method switching can be indicative of low satisfaction with the method used or poor service delivery. On the other hand, lower levels of method switching could mean user choices are limited [3].

The result from logistic regression analysis showed that women whose age ranged from 25–29 years were three to four fold less likely to switch method. Women who were 30–34 years of age were four times less likely to switch compared to those who were 15–19 years, and women who were 35 and above were six times less likely to switch compared to those women who were 15–19 years old. Similar study in Indonesia showed that as age increases, the probability of method switching decreases; those in the age group of 30–39 years had a low probability of switching method compared to their counterparts [20].

Married women were two times less likely to switch method compared to unmarried ones. In contrast to this study, studies in the Northern Africa indicated that method switching was common among married women than unmarried [21]. Women who had 2–4 children were three times less likely to switch long-acting method than those who had one child. In addition, women who had more than five children were two times less likely to switch long-acting method than those who had one child. Similar study from Indonesia in 2010 showed that women who want to stop birth were five times less likely to switch method compared to those who need additional children. This showed that women at higher parity were less likely to switch methods compared to women with no children [11, 12].

Source of family planning information has significant role in the use long-acting methods. Yet, if the information provided is not sufficient, the probability of switching will be high. In this study, women who heard information about family planning from health care providers were less likely to switch method compared to women who received from other people. A health professional gives sufficient information on the methods, hence, continuity of its use is better [7].

Majority of FGD participants reported that side effects were the primarily reasons for shifting methods. “*I have four children and I decided to take family planning then the health care provider convinced me to take long term contraceptives and their advantages. But I couldn’t tolerate excessive bleeding and and becoming weak and tired, that pushed me to change the method”-----28 year old woman* [Sabian Health Center]

*“After insertion of IUD, my menstruation became twice in a month and problem of stomachache became a common phenomenon, hence, it appeared very difficult to continue with it”.* —*35year old* [Melka health center]*.*

*“Since the time I put implant in my arm, I had seen no menstruation at all. I am afraid if blood accumulates in my abdomen, it can cause presser. So, I decided to take the implant out and shift to pills.” --------22 year old woman -* [Legeharie health center]*.*

Women whose husbands were not aware of their use of long-acting family planning method were less likely to switch from long-acting to short-acting method compared to those whose husbands were aware. But once they learnt of that their wives use of the method, they created problems on the family life and beyond; this led to premature termination of family planning method.

One FGD participant said, “*I have been using implant in my arm without my husband knowing about it, I was comfortable with the method. After a year he came to know that I am using this method and he went to the health facility and quarreled with the nurses. I was afraid thought that he may leave me and I might be left alone, so I terminated the method”….…30 year old woman* [Legeharie Health Center].

A qualitative study in Nigeria showed that, lack of support from the husband does not only hinder a woman’s decision to use a long-acting contraceptive methods, but it also may lead to premature termination of its use [22]. Husbands/partners, who do not support women on the use of family planning, tend to have more children, and prevent their wives from using any family planning.

Some religious leaders discourage the use of family planning. This is true both in Christian and Muslim religions and fertility control discussions are not generally favored. In this study, few women reported that religious leaders advised them on the use of family planning. A study done in 2011 in Indonesia, where Islam is the dominant religion, method switching is not significantly associated with religion [23].

In this study, bleeding associated with the use of family planning and husband/partner not being aware of wife’s use of long-acting fertility control methods were the main reasons for method switching. Similar studies from developing countries reported the same kind of result whereby two-fifths of the women switched due to side effects and the remaining switched because their husbands disliked the methods [24, 25].

## Conclusion and Recommendation

Method switching from long-acting family planning to other method is high. Method switching is common among unmarried women, women who had one child, women who want to space children and women who were not sure of their future fertility, women who reported other than health care provider as a source of family planning information, and those who reported their husbands were not aware of their use of long-acting family planning methods. The main reason for method switching was adverse effects such as bleeding and numbness in the arm.

Authors would like to recommend, women should be advised properly at the start of family planning. The advice should focus on the method and its side effects, and intervention for problems related to the use. Health executive bodies should strengthen their effort in addressing the problems of women switching long-acting methods through effective IEC and BCC strategies.

## Abbreviations

AOR, adjusted odds ration; BCC, behavioral change communication; CI, confidence interval; COR, crude odds ratio; FGAE, Family Guidance Association of Ethiopia; FGD, focus group discussion; FHI, Family Health International; HEW, Health Extension Worker; HIV, human immuno virus; IEC, information education and communication; IRERC, Institutional Research Ethical Review Committee; IUCD, intrauterine contraceptive devices; MCH, Maternal and Chile Health; NGO, Non-Governmental Organization; OR, odds ratio; SD, standard deviation; STIs, sexually transmitted illnesses; USAID, United States AID.
